# Evaluation of enlarged laminectomy with lateral mass screw fixation in relieving nerve root symptoms and correcting kyphosis for cervical myelopathy and radiculopathy

**DOI:** 10.3389/fsurg.2023.1103804

**Published:** 2023-02-02

**Authors:** Zhao Fang, Yuqiao Li, Zongyu Huang, Gan Luo, Houzhi Yang, Haiyang Cheng, Tiantong Xu

**Affiliations:** ^1^ Department of Spine Surgery, Tianjin Union Medical Center, Tianjin, China; ^2^ Graduate School, Tianjin Medical University, Tianjin, China

**Keywords:** cervical myelopathy and radiculopathy, enlarged laminectomy, lateral mass screw fixation, kyphosis, anterior cervical corpectomy decompression and fusion

## Abstract

**Purpose:**

This study aimed to compare the surgical efficacy of enlarged laminectomy with lateral mass screw fixation (EL-LMSF) and anterior cervical decompression and fusion (ACDF) for multilevel cervical myelopathy and radiculopathy (CMR) related to kyphosis.

**Methods:**

75 patients were retrospectively reviewed and divided into ACDF and EL-LMSF group. Clinical results including operative time, blood loss, and postoperative complications were compared. The JOA scoring system was used to evaluate spinal cord function and the VAS score evaluate nerve root pain severity. Cervical alignment a C2–C7 was measured with Cobb method and compared to confirm the reconstruction effect.

**Results:**

Data on 75 patients (M/F: 41:34; EL-LMSF/ACDF:42/33) with the mean age of 57.5 years (range 43–72 year old) were reviewed retrospectively. Discectomy and/or sub-toal corpectomy in ACDF group was performed with a mean of 3.24 levels (range, 3–4). Enlarged laminectomy in EL-LMSF group was performed with a mean of 3.89 enlarged levels (range, 3–5). The procedure of ACDF group showed a shorter operation time (103 ± 22 min vs. 125 ± 37 min, *P* = 0.000) and less blood loss (78 ± 15 ml vs. 226 ± 31 ml, *P* = 0.000) compared than that of the EL-LMSF group. Patients treated with EL-LMSF indicated lower VAS for upper extremity (1.3 ± 1.7 vs. 3.3 ± 1.3, *P* = 0.003) and better curvature corrected (10.7 ± 4.2° vs. 8.5 ± 3.5°, *P* = 0.013). The difference were of statistical significance. No statistical difference was found after surgery in the JOA score (14.1 ± 1.7 vs. 13.5 ± 2.1, *P* = 0.222). During the follow-up period, 15.2% of patients in the ACDF group had complications including 2 cases with transient dysphagia, 1 case with C5 palsy, 1 case with axial pain, and 1 case with screw pullout 3 month after surgery. However, only 9.5% of cases in the EL-LMSF group experienced complications, including 3 cases of axial pain and 1 case of epidural hematoma.

**Conclusion:**

The EL-LMSF procedure requires a longer operation time and more blood loss because of the incision of the stenosed foramen. However, the procedure has obvious advantages in relieving nerve root symptoms and correcting cervical curvature with fewer postoperative complications.

## 1. Background

Coexisting cervical myelopathy and Radiculopathy (CMR) is a disabling and prevalent disease. The progression of inter-vertebral disc degeneration and cervical malalignment lead to spinal cord and nerve compression, and disruption of spine kinematics.

Surgical management for patients with multilevel CMR associated with kyphosis aims to decompress the spinal cord and nerve root and improve the sagittal alignment using either an anterior approach or a posterior approach. However, the optimal surgical procedure remains controversial. The procedure of anterior cervical decompression and fusion (ACDF) may be accompanied by a high incidence of fusion failure and adjacent segment degeneration especially for multilevel CMR associated with kyphosis (1). The traditional posterior Laminoplasty/laminectomy cannot relieve nerve root compression, and even causes stretching of the nerve root due to backward shifting of the spinal cord. Enlarged laminectomy with lateral mass screw fixation (EL-LMSF) seemed to be an alternative procedure (2). Enlarged laminectomy allows adequate decompression of the spinal cord and nerve root by widening the spinal canal dimensions and removing the posterior wall of the the stenosed inter-vertebral foramen (3). Posterior plating with lateral mass screws is in favor of correcting cervical curvature and providing stability.

This is a retrospective comparative study of two surgical procedures between EL-LMSF and ACDF procedure in the management of multilevel CMR with cervical kyphosis. The study is designed to investigate the surgical efficacy in decompressing nerve root and correcting cervical kyphosis.

## 2. Materials and methods

### 2.1. Ethics statement

Informed written consent was obtained from each patient. The study protocol was approved by the Ethics Committee of Tianjin Union Medical Center.

### 2.2. Patient population

All patients with multilevel CMR associated with kyphosis were consecutively screened at Tianjin Union Medical Center between 2017 and 2019. Inclusion criteria: (1) degeneration cervical spine disease; (2) more than 3 levels of compression of the spinal cord; (3) bilateral nerve root compression symptoms; (4) overall and/or segmental cervical kyphosis); (5) more than 1 year follow-up. Exclusion criteria: (1) previous history of cervical surgery, cervical tumor or trauma; (2) unilateral nerve root compression symptom; and (3) continuous ossification of the posterior longitudinal ligament.

A total 75 patients with complete dates were finally retrospectively reviewed and divided into ACDF and EL-LMSF group. Surgical choice to use corpectomy, discectomy, or hybrid decompression was depending on local compressive pathology and level. In EL-LMSF group, level of laminectomy and foraminotomy was confirmed according to the imaging study and clinical symptom.

### 2.3. Operative technique

#### 2.3.1. Enlarged laminectomy with lateral mass screw fixation (EL-LMSF)

**Figure 1:**
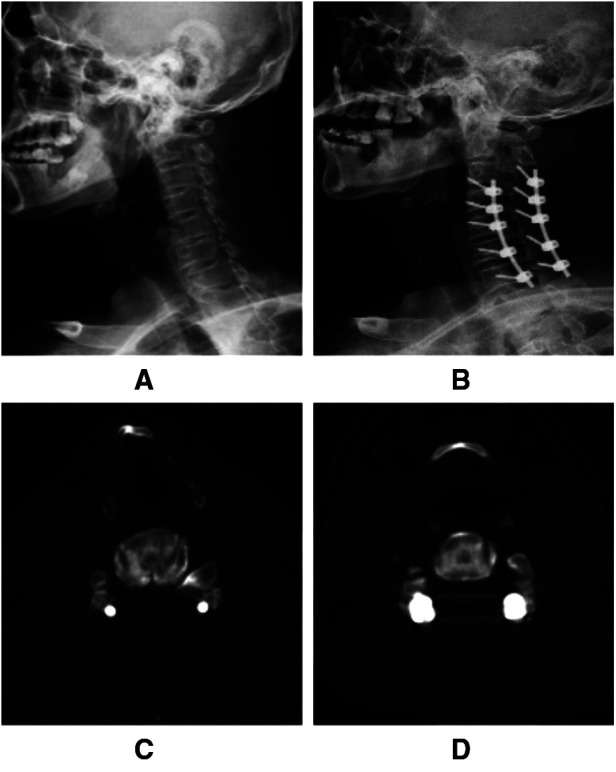
C3–C7 Enlarged laminectomy and lateral mass screw fixation with foraminotomy at the Lt. C4/5 and Rt. C4/5 and C5/6 levels. (**A,B**) Cervical curvature correction and foramen expanding at Rt. C4/5 and C5/6 levels. (**C,D**) Postoperative bilateral C4/5 and Lt. C5/6 foraminotomy.

The procedure was performed mainly in three steps as follows: lateral mass screw fixation, laminectomy and cutting the posterior wall of the intervertebral foramen. Posterior plating with lateral mass screw fixation were placed bilaterally in the modified Magerl's technique (4). Rods of appropriate size were selected and bent to match the contour of the lateral masses and secured to the lateral masses by screws, and then laminectomy were performed based on the preoperative surgical planning. While cutting the posterior wall of the inter-vertebral foramen, medial edge of the upper and lower facet resection should be ≤50%. This resection removed the posterior part of foramen thus making nerve root decompressed (Figure 1).

#### 2.3.2. Anterior cervical decompression and fusion with titanium plate and screw fixation (ACDF)

**Figure 2:**
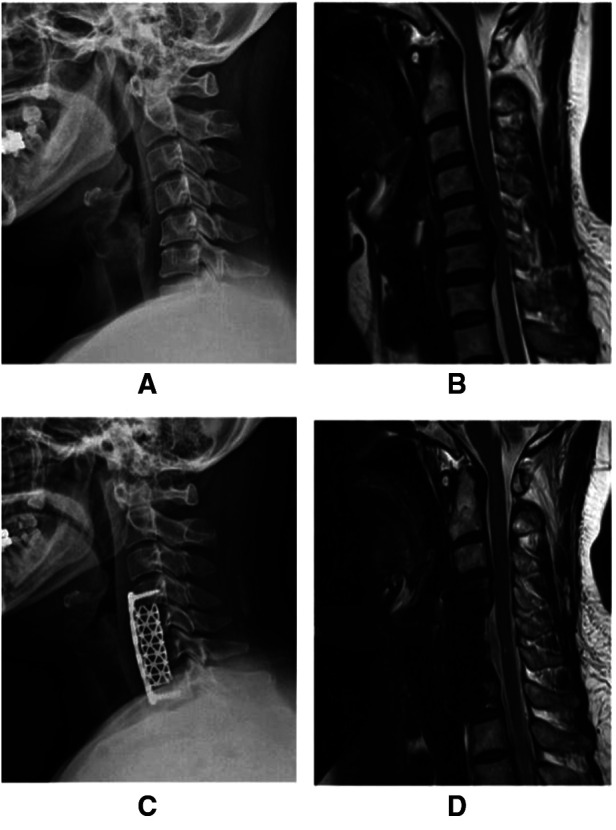
Anterior cervical subtotal corpectomy with titanium plate and screw fixation. (**A**) Lateral radiograph showing cervical kyphosis. (**B**) Posterior compressing of the spinal cord at the C4/5–C6/7 level. (**C**) Postoperative improved cervical alignment. (**D**) Postoperative MRI showing the decompressed spinal cord.

For the ACDF procedure, the removed discs and/or sub-total vertebrae were replaced by an appropriate-size cage or titanium mesh combined with small pieces of the bone allograft. The vertebrae above and the below fusion level were fixed with the appropriate-sized titanium plate and screw. The positions of cage, plate and screws were confirmed with C-arm (Figure 2).

### 2.4. Postoperative rehabilitation

Postoperative care was similar between the two groups. All patients wore a hard neck collar after surgery for 4–6 weeks. Pain management and some adjunctive therapy were emphasized equally. The wound drain tube was removed within 3 days after surgery. Patients were discharged from the hospital on the 7th postoperative day.

### 2.5. Clinical evaluation

The JOA (Japanese Orthopedic Association) scoring system was used to evaluate the severity of myelopathy based on the degree of dysfunction in each category. The VAS (Visual Analog Scale) score was used to evaluate upper extremity pain caused by nerve root compression. The JOA and VAS score was investigated before surgery and on the postoperative 7th day, 3th month and 1st year.

Symptoms mentioned above consist of three categories: myelopathy (extremity weakness/numbness, gait instability, and bladder dysfunction), radiculopathy (upper extremity pain), and postoperative axial pain.

### 2.6. Radiographic measurements

Cervical alignments at C2–C7 was measured with Cobb method on lateral cervical radiograph (5). MR images were used to confirm the degree of spinal cord compression and postoperative extent, and CT scans to judge the placement of screw fixation. The radiographic measurements were performed by two independent surgeons before surgery and at the postoperative 1st year follow-up.

### 2.7. Statistical analysis

Unpaired *t*-tests or Mann–Whitney *U* tests were used to detect differences between the two groups. Statistical analyses were performed with SPSS 22.0 (SPSS, Inc., Chicago, IL, USA). *P* < 0.05 was considered to indicate statistical significance.

## 3. Results

### 3.1. Patients demographics

Data on 75 patients (M/F: 41:34; EL-LMSF/ACDF:42/33) with the mean age of 57.5 years (range 43–72 year old) were reviewed retrospectively. The average follow-up was from 14.8 to 55.2 months, and all of the follow-up dates were recorded completely at least within 1 year. The follow-up period between the two group was of no statistical difference. Discectomy and/or sub-toal corpectomy in ACDF group was performed with a mean of 3.24 levels (range, 3–4). Enlarged laminectomy in EL-LMSF group was performed with a mean of 3.89 enlarged levels (range, 3–5). The difference was no statistically significant. The level and side of foraminotomy include: C4/5 in 12 cases, C5/6 in 16 cases, C6/7 in 1 case, C4/5 and C5/6 in 9 cases, C5/6 and C6/7 in 4 cases. There was no statistical difference between the two groups for the preoperative JOA score, VAS for upper extremity and C2-C7 alignment (Table 1).

**Table 1:** Clinical characteristics of the patients before operation.

	ACDF group	EL-LMSF group	*P* value
Age	54.8 ± 11.7	57.2 ± 12.9	-
Male (%)	54.4%	56.2%	-
BMI (kg/m^2^)	23.1 ± 4.5	27.1 ± 5.1	0.078
JOA score	8.3 ± 2.0	7.9 ± 1.9	0.290
VAS score	7.2 ± 1.2	6.9 ± 1.3	2.084
C2–C7 angle	1.3 ± 9.3	0.7 ± 8.6	0.765

Mean ± SD; NS, not statistically significant; -, no statistical calculation; BMI, body mass index; JOA: Japanese Orthopedic Association; VAS, Visual Analog Scale.

### 3.2. Clinical results, radiographic results and postoperative complications

No statistical difference was found at 1 year-follow-up after surgery in the JOA score (14.1 ± 1.7 vs. 13.5 ± 2.1, *P* = 0.222) between the two procedures. The procedure of the ACDF group showed a shorter operation time (103 ± 22 min vs. 125 ± 37 min, *P* = 0.000) and less blood loss (78 ± 15 ml vs. 226 ± 31 ml, *P* = 0.000) compared to that of the EL-LMSF group. Patients treated with EL-LMSF indicated lower VAS for the upper extremity (1.3 ± 1.7 vs. 3.3 ± 1.3, *P* = 0.003) and better curvature corrected (10.7 ± 4.2° vs. 8.5 ± 3.5°, *P* = 0.013). The difference was of statistical significance.

In the EL-LMSF group, CT scans after surgery did not show any error in screw location, trajectory or length. While in the ACDF group, a patient was found with screw pullout 3 months after surgery, but the patient remains asymptomatic and the titanium mesh fuses solidly.

During the follow-up period, 15.2% of patients in the ACDF group had complications including 2 cases with transient dysphagia, 1 case with C5 palsy, and 1 case with axial pain. However, only 9.5% of cases in the EL-LMSF group experienced complications, including 3 cases of axial pain and 1 case of epidural hematoma (Table 2).

**Table 2:** Surgical results, radiology assessment and complications.

	ACDF group	EL-LMSF group	*P* value
**Clinical assessments**			
Operation time (min)	103 ± 22	125 ± 37	0.000
Blood loss (ml)	78 ± 15	226 ± 31	0.000
JOA score	14.1 ± 1.7	13.5 ± 2.1	0.222
VAS score	3.3 ± 1.3	1.3 ± 1.7	0.003
**Radiologic assessments**			
C2–C7 angle (°)	8.5 ± 3.5	10.7 ± 4.2	0.013
**Postoperative complications**			
Transient dysphagia	2	0	-
C5 palsy	1	0	-
Axial pain	1	3	-
Epidural hematoma	0	1	-
Screw pullout	1	0	-

Mean ± SD; *P* < 0.05: statistically significant difference; -, no statistical calculation; JOA: Japanese Orthopedic Association; VAS: Visual Analog Scale.

## 4. Discussion

### 4.1. Radiologic comparison in correcting cervical kyphosis

For patients with multilevel CMR with kyphosis, the aims of surgical treatment were not only decompressing the spinal cord and nerve root but also improving cervical alignment. Reconstruction of cervical alignment is essential in treating cervical radiculomyelopathy related to kyphotic deformity (6, 7). Poor reconstruction maybe offset the effect of decompression. Restoring alignment in conjunction with decompression has been purported to improve long-term patient-reported symptoms (8). Uchida et al. (9) confirmed adequate correction of sagittal alignment may help to maximize the chance of neurological improvement. For the anterior approach, the height and longitudinal diameter of the inter-vertebra can be increased with the help of implants like a cage or mesh (10). ACDF seems to be the mainstay procedure but at a limited number of levels. Multilevel anterior cervical corpectomy and fusion seem to be a radical surgical option because of the high incidence of fusion failure, titanium mesh subsidence, and adjacent segmental degeneration (1, 11). Duan et al. pointed out that posterior fixations could provide immediate stability to the cervical spine after laminectomy, correct cervical kyphosis and promote early neurological recovery (12). Up to date, there is seldom report about a comparison of radiographic results between EL-LMSF and ACDF procedures in the management of multilevel CMR with kyphosis.

At the last postoperative follow-up, the EL-LMSF group provided better cervical alignment than that of the ACDF group (10.7 ± 4.2° vs. 8.5 ± 3.5°, *P* = 0.013). The difference was of statistical significance. EL-LMSF procedure had an obvious advantage in correcting cervical curvature. During the follow-up period, there was no screw loose or curvature loss in EL-LMSF group, while a patient in ACDF group was found with screw pullout 3 months after surgery, but the patient remained asymptomatic and accepted regular follow-up.

### 4.2. Clinical comparison and postoperative complications

There was no statistical difference in the JOA score at the last postoperative follow-up between the two procedures (14.1 ± 1.7 vs. 13.5 ± 2.1, *P* = 0.222). While postoperative patients in the EL-LMSF group reported lower VAS scores for upper extremity pain than that of the ACDF group (1.3 ± 1.7 vs. 3.3 ± 1.3, *P* = 0.003), which indicated patients with EL-LMSF procedure received better treatment effect in relieving nerve root symptoms. During the follow-up period, 15.2% of patients developed postoperative complications in the ACDF group, while 9.5% of patients were in the EL-LMSF group. We still believe EL-LMSF procedure is more safe than ACDF especially for more than 4-level stenosis lesion, Although axial pain was an unavoidable complication after posterior surgery.

As we know, the longitudinal diameter of inter-vertebral foramen was increased with the help of implants. While anteroposterior diameter was widen in the way of excision of uncovertebral joint or facet joint (13). The Choice of surgical procedure for radiculopathy should be based on the foraminal stenosis mechanism. The neural foramen is a funnel-shaped structure where the nerve root extending from the spinal cord is the widest and the root localization is the narrowest. This anatomical characteristic provides evidence for enlarged laminectomy (14). The technique of foraminotomy has been continually developed since the 1990s with a satisfying result of decompressing the nerve root and preventing nerve root paralysis (15). The reported incidence of postoperative C5 palsy was 4.6% (range from 0% to 30%) (16). In this study, the ACDF group had a patient with C5 palsy, whereas none of the patients in the EL-LMSF group experienced this condition. This result demonstrates that an enlarged laminectomy can prevent postoperative C5 palsy, and many other studies have reported similar findings (17, 18).

Axial pain has been defined as pain from the nuchal to the periscapular or shoulder region (19). Such a complication has been reported mainly after posterior cervical surgery. The underlying mechanisms of axial pain are still not fully understood. Potential sources of axial pain include posterior muscle atrophy, the muscles detaching from C2 or C7, and sinking or nonunion of the expanded laminae (20, 21). The decrease in cervical lordosis or increase in cervical kyphosis would produce more axial pain. It is reported that reconstruction of the posterior tension band decreased the incidence of axial pain (22). The incidence of axial symptoms after laminectomy or laminoplasty can be as high as 30%. In this study, 7.1% of patients complained of postoperative axial pain which occurred mainly in patients with obvious cervical kyphosis. The decreasing incidence of axial symptoms may correlate with the reconstruction of posterior tension band and restoration of cervical curvature. This finding is consistent with the findings of previous reports (23, 24). However, few high-quality clinical trials have reported this association.

In conclusion, the EL-LMSF procedure requires a longer operation time and more blood loss because of the incision of the stenosed foramen. However, the procedure has obvious advantages in relieving nerve root symptoms and correcting cervical curvature with fewer postoperative complications. However, Large scale clinical trials with long-term follow-up are urgently warranted. We expect a further correlation analysis between the reconstruction of alignment and neurological function.

## Data Availability

The raw data supporting the conclusions of this article will be made available by the authors, without undue reservation.
